# Supplementary material for: The In Vitro Pharmacokinetics of Medicinal Plants: A Review
Source: Pharmaceuticals (Basel). 2025 Apr 9;18(4):551. doi: 10.3390/ph18040551 (PMC12030146; doi:10.3390/ph18040551)
Supplement: Supplementary file 1 [file pharmaceuticals-18-00551-s001.zip › pharmaceuticals-3500670-supplementary.pdf]

Table S1 - MeSH/PubMed search strategy

PUBMED	"(""Medicinal Plant""[Title/Abstract] OR ""plant medicinal""[Title/Abstract] OR ""Medicinal Plants""[Title/Abstract] OR ""Medicinal Herbs""[Title/Abstract] OR ""herb medicinal""[Title/Abstract] OR ""Medicinal Herb""[Title/Abstract] OR ""herbs medicinal""[Title/Abstract] OR ""Pharmaceutical Plants""[Title/Abstract] OR ""Pharmaceutical Plant""[Title/Abstract] OR ""plant pharmaceutical""[Title/Abstract] OR ""plants pharmaceutical""[Title/Abstract] OR ""Healing Plants""[Title/Abstract] OR ""Healing Plant""[Title/Abstract] OR ""plant healing""[Title/Abstract] OR ""plants healing""[Title/Abstract]) AND (""In Vitro Technique""[Title/Abstract] OR ""technique in vitro""[Title/Abstract] OR ""techniques in vitro""[Title/Abstract] OR ""In Vitro""[Title/Abstract] OR ""In Vitro Testing""[Title/Abstract] OR ""In Vitro Testings""[Title/Abstract] OR ""testing in vitro""[Title/Abstract] OR ""vitro testing in""[Title/Abstract] OR ""In Vitro Test""[Title/Abstract] OR ""test in vitro""[Title/Abstract] OR ""tests in vitro""[Title/Abstract] OR ""In Vitro Tests""[Title/Abstract]) AND (""Drug Kinetics""[Title/Abstract] OR ""kinetics drug""[Title/Abstract] OR ""ADME""[Title/Abstract] OR ""Absorption""[Title/Abstract] OR ""Distribution""[Title/Abstract] OR ""Metabolism""[Title/Abstract] OR ""Elimination""[Title/Abstract])"
WEB OF SCIENCE	"TS=(“Medicinal Plant” OR “Plant, Medicinal” OR “Medicinal Plants” OR “Medicinal Herbs” OR “Herb, Medicinal” OR “Medicinal Herb” OR “Herbs, Medicinal” OR “Pharmaceutical Plants” OR “Pharmaceutical Plant” OR “Plant, Pharmaceutical” OR “Plants, Pharmaceutical” OR “Healing Plants” OR “Healing Plant” OR “Plant, Healing” OR “Plants, Healing”)" AND "TS=(“In Vitro Technique” OR “Technique, In Vitro” OR “Techniques, In Vitro” OR “In Vitro” OR “In Vitro Testing” OR “In Vitro Testings” OR “Testing, In Vitro” OR “Vitro Testing, In” OR “In Vitro Test” OR “Test, In Vitro” OR “Tests, In Vitro” OR “In Vitro Tests”)" AND "TS=(“Drug Kinetics” OR “Kinetics, Drug” OR “ADME” OR “Absorption” OR “Distribution” OR “Metabolism” OR “Elimination”)"
SCOPUS	(ABS ("Medicinal Plant" OR "Plant, Medicinal" OR "Medicinal Plants" OR "Medicinal Herbs" OR "Herb, Medicinal" OR "Medicinal Herb" OR "Herbs, Medicinal" OR "Pharmaceutical Plants" OR "Pharmaceutical Plant" OR "Plant, Pharmaceutical" OR "Plants, Pharmaceutical" OR "Healing Plants" OR "Healing Plant" OR "Plant, Healing" OR "Plants, Healing") AND ABS ("In Vitro Technique" OR "Technique, In Vitro" OR "Techniques, In Vitro" OR "In Vitro" OR "In Vitro Testing" OR "In Vitro Testings" OR "Testing, In Vitro" OR "Vitro Testing, In" OR "In Vitro Test" OR "Test, In Vitro" OR "Tests, In Vitro" OR "In Vitro Tests") AND ABS ("Drug Kinetics" OR "Kinetics, Drug" OR "ADME" OR "Absorption" OR "Distribution" OR "Metabolism" OR "Elimination"))
